# A comprehensive review of small regulatory RNAs in *Brucella* spp.

**DOI:** 10.3389/fvets.2022.1026220

**Published:** 2022-12-01

**Authors:** Kellie A. King, Mitchell T. Caudill, Clayton C. Caswell

**Affiliations:** Center for One Health Research, Department of Biomedical Sciences and Pathobiology, VA-MD College of Veterinary Medicine, Virginia Tech, Blacksburg, VA, United States

**Keywords:** *Brucella* species, small RNAs (sRNAs), virulence, intracellular bacteria, *Alphaproteobacteria*

## Abstract

*Brucella* spp. are Gram-negative bacteria that naturally infect a variety of domesticated and wild animals, often resulting in abortions and sterility. Humans exposed to these animals or animal products can also develop debilitating, flu-like disease. The brucellae are intracellular pathogens that reside predominantly within immune cells, typically macrophages, where they replicate in a specialized compartment. This capacity of *Brucella* to survive and replicate within macrophages is essential to their ability to cause disease. In recent years, several groups have identified and characterized small regulatory RNAs (sRNAs) as critical factors in the control of *Brucella* physiology within macrophages and overall disease virulence. sRNAs are generally < 300 nucleotides in length, and these independent sRNA transcripts are encoded either next to (i.e., *cis*-encoded) or at a distant location to (i.e., *trans*-encoded) the genes that they regulate. *Trans*-encoded sRNAs interact with the mRNA transcripts through short stretches of imperfect base pairing that often require the RNA chaperone Hfq to facilitate sRNA-mRNA interaction. In many instances, these sRNA-mRNA interactions inhibit gene expression, usually by occluding the ribosome-binding site (RBS) and/or by decreasing the stability of the mRNA, leading to degradation of the transcript. A number of sRNAs have been predicted and authenticated in *Brucella* strains, and a variety of approaches, techniques, and means of validation have been employed in these efforts. Nonetheless, some important issues and considerations regarding the study of sRNA regulation in *Brucella* need to be addressed. For example, the lack of uniform sRNA nomenclature in *Brucella* has led to difficulty in comparisons of sRNAs across the different *Brucella* species, and there exist multiple names in the literature for what are functionally the same sRNA. Moreover, even though bona fide sRNAs have been discovered in *Brucella*, scant functional information is known about the regulatory activities of these sRNAs, or the extent to which these sRNAs are required for the intracellular life and/or host colonization by the brucellae. Therefore, this review summarizes the historical context of Hfq and sRNAs in *Brucella*; our current understanding of *Brucella* sRNAs; and some future perspectives and considerations for the field of sRNA biology in the brucellae.

## Review

*Brucella* species naturally infect a wide range of wild and domesticated animals, including cattle, goats, and swine. In these animals, infection results in the disease brucellosis, which is often characterized by reproductive pathologies, such as abortions and sterility (1). *Brucella* species are additionally zoonotic pathogens, as they are efficiently transmitted from animals to humans, and the resulting infections are characterized by a debilitating, flu-like illness that often presents with a characteristic relapsing fever (2). While rare, complications of chronic human brucellosis can be fatal due to infective endocarditis. More common, non-lethal symptoms, such as arthritis and neurological symptoms, also contribute to the morbidity of humans suffering from chronic brucellosis (3). Additionally, given their small size, low infectious dose, and easy aerosolization, some *Brucella* species are considered potential agents of biological weaponry (4).

As the brucellae are intracellular pathogens that replicate in specialized niches within mesenchymal cells, they must carefully coordinate expression of genes encoding numerous metabolic, virulence-associated, and cell division proteins to successfully persist within their hosts (5). Despite the importance of *Brucella* temporo-spatially regulating transcription and translation in order to replicate and result in virulent brucellosis, small regulatory RNAs (sRNAs) remain poorly characterized within the brucellae. Here, we review the identified and confirmed sRNAs within *Brucella*, discuss their known or speculated connection with development of virulent brucellosis, and outline a few of the critical questions that remain to be addressed regarding sRNAs and *Brucella*.

### What are small RNAs?

Bacterial small regulatory RNAs (sRNAs) are single-stranded RNA molecules that range between 50 and 300 nucleotides long. sRNAs are conceptually divided into two distinct classes, *cis-*encoded and *trans-*encoded, based on their genetic location in relationship to their functional site (6). *Cis-*encoded sRNAs are encoded on the DNA strand immediately opposite of their regulatory target and engage in extensive RNA-RNA complementary base-pairing with the mRNA transcript of the regulated protein. These sRNA-mRNA interactions commonly serve to inhibit protein translation through occlusion of ribosome binding (7). *Trans-*encoded sRNAs are encoded in an area of the genome independent of their regulatory target(s), and are often located between protein-encoding open reading frames. *Trans*-encoded sRNAs typically act through short segments known as seed-regions that form in the secondary structure of the sRNA (8, 9). The exposed single strand of nucleotides in the seed region can then bind with mRNA target, resulting in the regulation. Such interactions between *trans*-encoded sRNAs and their mRNA targets are often mediated by the RNA chaperone protein Hfq, which is discussed more extensively below (10).

*Trans*-encoded sRNA regulation can either positively or negatively impact protein translation. Some *trans*-encoded sRNAs increase protein levels through stabilization of the mRNA transcript through inhibition of RNase activity resulting in less transcript turnover and more protein translation or by restructuring the mRNA transcript to reveal the ribosome binding site (11). Alternatively, sRNA binding can negatively impact gene expression. For instance, the sRNA-mRNA binding can create a double-stranded RNA molecule that is targeted for degradation by RNases, or restructures the mRNA transcript to occlude the ribosome binding site and thus inhibit translation (6). Regardless of the exact mechanism, in the archetypical scenario sRNAs act as a layer of post-transcriptional regulation to fine-tune protein translation.

### Hfq: The riboregulator

First described as a host-factor of *Escherichia coli* required for the replication of the RNA Qβ phage, Hfq has proven to be an RNA binding protein deeply conserved across bacteria (12). Hfq is also considered a major virulence factor in many bacteria, as Δ*hfq* mutants exhibit pleiotropic phenotypes, including delayed growth rate, small colony phenotypes, increased sensitivities to a variety of stresses, and decreased virulence (13–16). Structurally, Hfq is an Sm-like family protein that is amazingly found in all three domains of life, showcasing the evolutionary role this RNA chaperone encompasses (17–19). Hfq forms a homohexamer structure that reveals 3 binding faces: the distal face, the proximal face, and the outer ring or rim surface (20). Each binding face has specific affinity for properties of either sRNAs or mRNAs. The distal face of Hfq preferentially binds single stranded ARN or ARNN motifs, (R signifies a purine and N represents any base), commonly found on mRNA sequences. The proximal face has high affinity for RNA molecules enriched with Uracil residues, which are commonly found in Rho-independent terminator sequences of sRNAs, followed by stem loop structures (21). The rim or lateral side of Hfq was recently found to be another important interaction face that binds UA-rich RNA molecules through positively charged residues (22). Hfq facilitates RNA regulation by increasing local concentrations of sRNA and mRNA targets, and can induce structural changes to assist in sRNA binding (23). By spatially concentrating sRNA and mRNAs, Hfq facilitates the imperfect base-pairing and regulatory actions of the sRNA.

**Figure 1:**
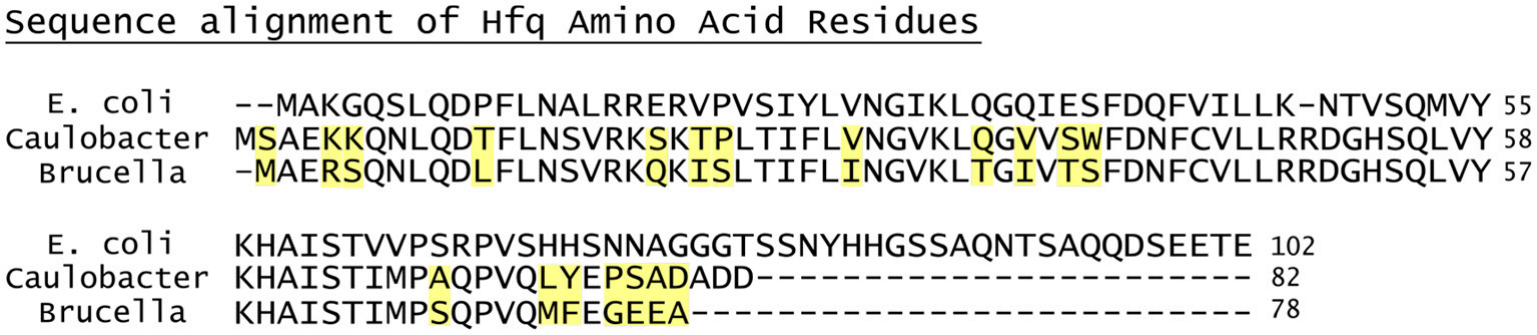
The protein sequence alignment of Hfq for *Escherichia coli, Caulobacter crescentus* and classical *Brucella* species. Amino acid differences between *C. crescentus* and *Brucella* are highlighted in yellow. Alignment was performed in the UniProt platform using the Clustal Omega algorithm (26, 27).

It is important to note that many of biophysical properties of Hfq have been determined from studies involving *Gammaproteobacteria*, such as *Escherichia coli*. Comparative studies of the Hfq of *E. coli* to that of *Caulobacter crescentus*, an alphaproteobacterium more closely related to *Brucella*, reveals significant biophysical differences between *E. coli* and *C. crescentus* in Hfq-RNA interactions at the C-terminal domain (24, 25). Hfq proteins in *Brucella* strains discussed in this review (i.e., *B. abortus, B. melitensis*, and *B. suis*) are 100% identical at the amino acid level, and interestingly, Hfq proteins in both *C. crescentus* and *Brucella* are approximately 20% shorter than the *E. coli* Hfq protein due to truncations in the C-terminal portion of the proteins (Figure 1). These differences in the C-terminal regions of Hfq in *C. crescentus* and *E. coli* are responsible for the distinctive binding properties of each protein, but to date, careful biophysical studies of the *Brucella* Hfq protein have not been performed (24, 25). Additionally, the Hfq protein of *Brucella* spp. has not yet been characterized by either crystallography or cryoelectron microscopy to allow molecular docking analysis. Given the amino acid conservation, we would tentatively suggest that the *Brucella* Hfq acts in a manner more similar to that of *C. crescentus* than *E. coli*, but this remains speculative until further data are available.

Nonetheless, the variability of Hfq biophysical binding characteristics and amino acid sequence differences underscore the evolutionary specialization of Hfq proteins to fine-tune bacterial regulatory networks to meet changing environments. This observation is borne out in the links between Hfq and virulence in numerous bacteria from across multiple orders, including *Pseudomonas aeruginosa* (13), *Vibrio cholerae* (15), *Salmonella typhimurium* (28), *Francisella tularensis* (29), *Neisseria meningitidis* (30), *Yersinia pestis* (14), and *Yersinia pseudotuberculosis* (31). Hfq is additionally important for the successful colonization of hosts in other *Alphaproteobacteria*, such as the plant symbiont *Sinorhizobium* (32) or plant pathogen *Agrobacterium* (33). Importantly, the link between Hfq and bacterial virulence was first described *B. abortus*, as a *B. abortus* Δ*hfq* strain was shown to be highly attenuated in both cellular and animal models of infection (16).

### sRNAs in *Brucella*

*Brucella* is an intracellular pathogen that resides within macrophages and dendric cells in a specialized compartment called the *Brucella*-containing vacuole (BCV) (34, 35). The BCV is trafficked through the macrophage where the bacteria are exposed to a variety of stresses and signals, and the brucellae must sense and adapt to these harsh conditions in order to survive and replicate within the host cells (5). Central to the adaptation of *Brucella* species to their intracellular environment is the use of sRNAs to quickly and efficiently alter gene expression and protein levels in order to cope with the onslaught of cellular defense mechanisms, as outlined in the following sections. Before proceeding to the discussion of sRNAs, it is important to note that the nomenclature and species status within the genus *Brucella* is fairly unique. The debates regarding *Brucella* nomenclature have been expertly reviewed elsewhere (36, 37). The three *Brucella* species examined in this review, *B. abortus, B. melitensis*, and *B. suis*, are approximately 97% similar at the nucleotide level and show a closed pangenome (38–40). As such, while we present and discuss the sRNAs in the particular species in which they were identified, it is highly likely that a sRNA found in one species is also produced in the other two species, and we will note specific cases where this cross-species conservation has been experimentally validated.

The first sRNAs confirmed in *B. abortus* were called AbcR1 and AbcR2 due to their homology to the *Agrobacterium tumefaciens* sRNAs, AbcR1 and AbcR2, which exhibit regulatory activities toward genes encoding of ABC-type transport systems (41). These sRNAs are conserved among many members of the *Rhizobiales*, which includes bacteria such as *Agrobacterium* spp., *Sinorhizobium* spp., and *Brucella* spp. (42). The AbcR sRNAs were identified in a study analyzing a *B. abortus hfq* deletion strain, and in that study, two proteins, BAB1_1794 and BAB2_0612, were shown to be highly over-produced when *hfq* was deleted (43). These two proteins are orthologs of the *A. tumefaciens* proteins Atu2422 and Atu1879, respectively, and it had been reported that the production of Atu2422 and Atu1879 was regulated by AbcR1 and AbcR2 in *A. tumefaciens* (41). Bioinformatic analyses revealed that potential AbcR1 and AbcR2 orthologs were encoded in the *B. abortus* genome, and subsequent northern blot analyses determined that bona fide AbcR sRNAs were indeed produced by *B. abortus* (43). Importantly, the AbcR sRNAs are fully conserved in *B. abortus* 2308, *B. melitensis* 16M, and *B. suis* 1330, as northern blot analyses demonstrated that all three strains produce AbcR1 and AbcR2. Mutational analyses revealed that AbcR1 and AbcR2 are together essential for *Brucella*'s virulence in macrophages and in a mouse model of infection. Deletion of either *abcR* gene individually did not alter the virulence of *B. abortus*, but deletion of both *abcR1* and *abcR2* resulted in significant attenuation in both macrophages and mice. These data indicated that AbcR1 and AbcR2 perform redundant functions. In order to identify the regulatory targets of the AbcR sRNAs in *B. abortus*, transcriptomic and proteomic analyses were performed, and these experiments demonstrated that 25 transcripts were over-expressed and 16 proteins over-produced in the *abcR1 abcR2* deletion strain compared to the parental strain. It was shown that deletion of *abcR1* and *abcR2* leads to increased stability of target mRNAs, indicating that the AbcR sRNAs facilitate the degradation of these mRNAs. Of note, the genes exhibiting dysregulation in the *abcR1 abcR2* mutant encode components of ABC-type transport systems, including BAB1_1794, BAB2_0612, and BAB2_0879. The periplasmic-binding proteins BAB1_1794 and BAB2_0879 are part of transport systems involved in the importation of the non-proteogenic amino acid GABA in *B. abortus*, but neither protein is required for the full virulence of *B. abortus* (44, 45). While the function of BAB2_0612 remains unknown, it is clear that BAB2_0612 is required for *B. abortus* virulence, as a *bab2_0612* deletion strain is highly attenuated in a mouse model of infection (45).

Following the initial characterization of the *Brucella* AbcR sRNAs, a mechanistic study defined the specific elements of AbcR1 and AbcR2 that control the expression of specific subsets of genes, as well as virulence (45). It was shown that two distinct regulatory motifs, called M1 and M2, are present in both AbcR1 and AbcR2, but strikingly, the M2 motif is alone responsible for controlling the virulence-linked gene expression pathways of the AbcR sRNAs. The M1 and M2 seed regions, CUCCCA and GUUCCC, respectively, interact with complementary sequences in target mRNAs, leading to degradation of the mRNAs, and direct binding of AbcR1 and AbcR2 to the BAB1_0879 mRNA was experimentally validated. Interestingly, the M2-targeted BAB2_0612 mRNA, but not BAB1_0879, is required for the full virulence of *B. abortus*. As noted above, the physiological activity of BAB2_0612 is currently unknown, but it is clear that AbcR-regulated BAB2_0612 by means of the M2 seed region is required for *B. abortus* virulence. Regarding the M1 and M2 motifs, these regulatory regions are entirely conversed in the AbcR1 and AbcR2 sRNAs of *B. abortus, B. melitensis* and *B. suis*. While the AbcR sRNA regulatory circuit has been illuminated in recent years, there are still several important questions that remain about this important pathway. First, the identity of a transcriptional regulator of *abcR1* expression has not been determined. Sheehan et al. identified the LysR-type transcriptional regulator, VtlR, as an activator of *abcR2* expression, and VtlR is crucial for *B. abortus* virulence (56). However, the transcriptional regulator of *abcR1* is not known. Is *abcR1* constitutively expressed, or is *abcR1* expression under the control of a specific regulator or regulators? Second, the RNase (or RNases) involved in AbcR-mediated degradation of target mRNAs has not been described. Specific RNases have been studied in *Brucella* strains, but to date, none of the studied RNases have been linked to AbcR regulatory events (53, 55). Finally, what are the dynamics of *abcR* expression during infection of macrophages and animals? This information will be extremely valuable for the complete elucidation of the mechanism(s) of AbcR-linked virulence in *Brucella*.

Since the initial discovery of sRNAs in *Brucella*, many bioinformatics prediction programs have been implemented to search for additional sRNAs. Many pipelines have predicted sRNAs in several different *Brucella* species. Although many of these findings remain as predictions, some sRNA candidates have been validated, but very few have been functionally characterized. Regarding prediction vs. authentication, this review focuses on *Brucella* sRNAs whose existence have been confirmed using northern blot analysis (i.e., the gold standard) or RT-PCR analysis, and a summary of bona fide *Brucella* sRNAs is reported in Table 1. While many of the prediction approaches and large-scale transcriptomic analyses provide vast lists of putative sRNAs, rigorous validation experiments are needed to substantiate the authenticity of these sRNAs.

**Table 1:** Confirmed *Brucella* sRNAs summarized in this review.

**Name of sRNA**	***Brucella* species**	**Orientation**	**Flanking genes**	**Predicted Size (nt)**	**Confirmed with?**	**Citation**
AbcR1	*B. abortus*	→ ← →	BAB_RS28805-BAB_RS28810	116	Northern	(43)
AbcR2	*B. abortus*	→ ← →	BAB_RS23130-BAB_RS23145	110	Northern	(43)
BASRCI408	*B. abortus*	→ ← →	BAB_RS19990-BAB_RS19995	434	RT-PCR	(46)
BASRCI27	*B. abortus*	→ → →	BAB_RS16650-BAB_RS16655	166	T-PCR	(46)
BASRCI385	*B. abortus*	← → ←	BAB_RS18175-BAB_RS18180	204	RT-PCR	(46)
BASRCI337	*B. abortus*	← → ←	BAB_RS25025-BAB_RS25030	190	RT-PCR	(46)
BASRCI414	*B. abortus*	← → ←	BAB_RS20005-BAB_RS20010	228	RT-PCR	(46)
BASRCI153	*B. abortus*	→ → →	BAB_RS23010-BAB_RS234265	213	RT-PCR	(46)
BsrH	*B. abortus*	← → ←	BAB_RS26720-BAB_RS26725	150	RT-PCR	(47)
BAS I 74	*B. abortus*	Unknown	BAB_RS32375-BAB_RS19410	86	RT-PCR	(48)
BSR0602	*B. melitensis*	← → ←	BME_RS13135-BME_RS16140	169	Northern	(49)
BSR0709	*B. melitensis*	←↔ →	BME_RS13690-BME_RS13680	317	Northern	(49)
BSR0653	*B. melitensis*	→ ← →	BME_RS13385-BME_RS13380	642	Northern	(49)
BSR1350	*B. melitensis*	←← →	BME_RS06800-BME_RS06790	176	Northern	(49)
BSR0739	*B. melitensis*	← → →	BME_RS03670-BME_RS03675	160	Northern	(49)
BSR1073	*B. melitensis*	← → ←	BME_RS15395-BME_RS15405	196	Northern	(49)
BSR0626	*B. melitensis*	← → ←	BME_RS13240-BME_RS13245	128	Northern	(49)
BSR1141	*B. melitensis*	← → ←	BME_RS05715-BME_RS05720	198	Northern	(49, 50)
BSnc115	*B. suis*	→ ← →	BR_RS01535-BR_RS01540	91	Northern	(51)
BSnc118	*B. suis*	→ ← →	BR_RS03790-BR_RS03795	152	Northern	(51)
BSnc119	*B. suis*	→ ← →	BR_BS04580-BR_RS04585	90	Northern	(51)
BSnc120	*B. suis*	→ ←←	BR_RS04640-BR_RS04645	89	Northern	(51)
BSnc121	*B. suis*	←← →	BR_RS05430-BR_RS05435	116	Northern	(51)
BSnc135	*B. suis*	←←←	BR_RS07890-BR_RS07895	91	Northern	(51)
BSnc140	*B. suis*	←← →	BR_BS08505-BR_RS08510	94	Northern	(51)
BSnc149	*B. suis*	← → ←	BR_RS12945-BR_RS12950	155	Northern	(51)
BSnc150	*B. suis*	→ → →	BR_RS15180-BR_RS15185	44	Northern	(51)
BSnc159	*B. suis*	← → ←	BR_RS10950-BR_RS18055	52	Northern	(51)
BSR0441	*B. melitensis*	← → ←	BME_RS17155-BME_RS12365	250	RT-PCR	(52)
BM-sr00117	*B. melitensis* (027)	Unknown	Unknown	Unknown	Northern	(53)
Bmsr1	*B. melitensis* (M28)	→ → ←	BME_RS01840-BME_RS01835	121	Northern	(54)
Bmsr2	*B. melitensis* (M28)	← → →	BME_RS02410-BME_RS02405	244	Northern	(54)
Bmsr3	*B. melitensis* (M28)	→ → →	BME_RS05285-BME_RS05280	123	Northern	(54)
Bmsr4	*B. melitensis* (M28)	← → →	BME_RS00940-BME_RS00935	167	RT-PCR	(54)
Bmsr5	*B. melitensis* (M28)	← → ←	BME_RS04365-BME_RS04360	129	RT-PCR	(54)
Bmsr6	*B. melitensis* (M28)	← → →	BME_RS06570-BME_RS06565	166	RT-PCR	(54)
Bmsr7	*B. melitensis* (M28)	← → ←	BME_RS03710-BME_RS03705	152	RT-PCR	(54)
Bmsr8	*B. melitensis* (M28)	→ ← →	BME_RS05140-BME_RS05135	156	RT-PCR	(54)
Bmsr9	*B. melitensis* (M28)	← → ←	BME_RS04415-BME_RS04405	131	Northern	(54)
Bmsr10	*B. melitensis* (M28)	← → →	BME_RS13685-BME_RS13680	181	Northern	(54)
Bmsr11	*B. melitensis* (M28)	→ ← →	BME_RS15405-BME_RS15385	169	RT-PCR	(54)
Bsr4	*B. abortus*	→ ← →	BAB_RS19940-BAB_RS19945	124	Northern	(55)

Dong et al. (46) used the bioinformatics program SIPHT to search for putative rho-independent terminators within intergenic regions (i.e., known sRNA signature elements of many bacterial sRNAs), paired with the NAPP database (http://rssf.i2bc.paris-saclay.fr/NAPP/index.php), which predicts bacterial sRNA elements, to predict 129 sRNA candidates that were limited to intergenic regions (46, 57). These sRNA candidates were organized using a BASRC nomenclature for *B. abortus* sRNA candidate followed by chromosome number and candidate number. Of these 129 sRNA candidates, only 7 out of 20 were verified using RT-PCR. The regulatory targets of those 7 sRNAs were predicted using another bioinformatics software, sTarPicker (58). The predicted sRNA-target regulatory interactions were then tested using a two-plasmid *lacZ* reporter system in *E. coli*. This is a commonly used approach in the field that relies on a low-copy plasmid carrying a gene reporter (e.g., *GFP, lacZ*) that is fused to the regulatory region (e.g., 5'-untranslated region) of a putative sRNA-targeted gene, and a high-copy plasmid from which the gene encoding the sRNA of interest is expressed (reference to one prominent system used in the field: (59)). Using this type of system, the activity of the gene reporter can be monitored in the presence and absence of the sRNA to gain insight into the regulatory activity of a sRNA on a specific target mRNA. In the study by 44, the two-plasmid reporter system demonstrated that expression of BASRCI408, BASRCI385, BASRCI414, BASRCI153, or BASRCII26 led to reduced β-galactosidase activity when *lacZ* was fused to BAB1_2002, BAB1_0472, BAB1_0854, BAB1_1361, and BAB2_0187, respectively, indicating that these sRNAs negatively regulate their specific target genes. In the end, further investigation on the regulatory mechanism of these sRNAs is needed to gain insight into the regulatory activities these sRNAs, and importantly, more work is needed to understand the role of these sRNAs in *Brucella* physiology and virulence.

The Dong et al. (46) study led to the further characterization of one sRNA candidate that, after further characterization, was named BsrH for *Brucella* sRNA regulating HemH (47). BsrH is a *cis-*encoded sRNA antisense of *hemH (bab2_0075* / *bab_rs26720)*, which encodes ferrochelatase, an enzyme involved in heme biosynthesis. Importantly, HemH is critical for the full virulence of *B. abortus* in cellular and mouse models of infection (60). The production of BsrH was validated using RT-PCR, and BsrH levels are increased in response to acidic and oxidative stress. The regulation of HemH by BsrH was tested using a two-plasmid reporter system in *E. coli*, and these experiments demonstrated that over-expression BsrH reduced β-galactosidase activity of a *hemH*-*lacZ* fusion, indicating that BsrH negatively regulates *hemH*. However, no direct binding studies between BsrH and HemH were reported. Even though BsrH controls the expression of *hemH*, over-expression of BsrH had no impact on the virulence of *B. abortus in vitro* or *in vivo*. Interestingly, in many *Gammaproteobacteria*, iron homeostasis is coordinated by sRNAs (e.g., RhyB, PrrF), but to date, analogous iron-linked sRNAs have not been identified in the *Alphaproteobacteria* (61, 62). As such, BsrH is a very exciting example of a potential connection between iron homeostasis and sRNAs in the *Alphaproteobacteria*, and more work is warranted to define BsrH in *Brucella*, as well as BsrH orthologs in other closely related bacteria.

In the previously described study by Dong et al. (46), the authors described the identification of 129 putative sRNAs, but in that study, only 7 (of 20 assessed) of the sRNAs were authenticated by RT-PCR. In a subsequent study, the authors verified another 43 sRNAs (of 109 tested) via RT-PCR from (48). Over-expression of one of these sRNAs, named BASI74 led to decreased survival in a macrophage model of infection. The target prediction program, sTarPicker, was used to predict regulatory targets, of which four mRNAs exhibited elevated levels when BASI74 was over-expressed in the wild-type background, indicating that BASI74 positively regulates these four genes: (BAB1_0847: hypothetical protein; BAB1_1154: SAM-dependent methyltransferase; BAB1_1335: hypothetical protein; and BAB1_1361: hypothetical protein) (58). However, the levels of the target mRNAs were unaffected in the *basI74* deletion strain. It is possible that BASI74 functions to control gene expression without interfering with the stead-sate levels of the mRNAs, as bacterial sRNAs can regulate the translation of mRNAs into proteins without affecting the stability of the mRNA (6). Overall, more work is needed to determine the regulatory and functional activities of BASI74 in *B. abortus*.

Wang *et al*. also employed another computational approach that limited the search to intergenic regions of the *B. melitensis* genome (49). Using RNAMotif and sRNAPredict programs to predict possible promoters and terminators within intergenic regions, 21 candidate *Brucella* sRNAs named BSR for *Brucella* small-noncoding sRNA followed by the gene number of the downstream protein-encoding gene were predicted (63, 64). Northern blot analyses were used to confirm 8 of the predicted sRNAs, and some of these confirmed sRNAs exhibited diminished levels in a *B. melitensis hfq* mutant, indicating that these sRNAs require Hfq for their stability. These sRNAs were: BSR0709, BSR0653, BSR1350, BSR0739, BSR1073, BSR0626, BSR0602, and BSR1141. One Hfq-dependent sRNA, BSR0602, showed the highest abundance during stationary phase of growth, and BSR0602 levels are elevated when the bacteria are exposed to acidic, oxidative, and heat stress. TargetRNA was used to predict targets of BSR0602, leading to the hypothesis that BSR0602 regulates the expression of BMEI0106, a global transcriptional regulator of the GntR family (65). qRT-PCR analyses revealed increased expression of BMEI0106 in a *bsr0602* deletion strain, and expression was almost abolished in a *bsr0602* overexpression strain. Additionally, a two-plasmid reporter system in *E. coli* confirmed the negative regulation of BMEI0106 by BSR0602, as well as demonstrated the motif within BSR0602 that binds to the *bmeI0106* mRNA. This study determined that a *bmeI0106* deletion strain is attenuated in a mouse model of infection, and while a *bsr0602* deletion strain exhibited similar infectivity as the wild-type strain, a *bsr0602* over-expression strain demonstrated decreased fitness compared to the wild-type strain in an *in vivo* competition experiment. These data indicate that the BSR0602 regulatory pathway is required for the full virulence of *B. melitensis*.

Wang et al. provided further characterization of BSR1141 in a subsequent report (50), and here, it was reported that BSR1141 expression is induced in response to stress conditions, including acidic and oxidative stress. In the same vein, a *bsr1141* deletion strain exhibits reduced survival in both oxidative and low pH environments, and this phenotype is genetically complemented when *bsr1141* is supplied *in trans* on a plasmid. Importantly, the *bsr1141* deletion strain is attenuated in experimentally infected mice, indicating that BSR1141 is required for *B. melitensis* virulence. Using bioinformatic approaches coupled with qRT-PCR analysis, it was determined that BSR1141 is linked to the proper expression of more than 40 individual genes; however, it is not clear how many of these putative targets are directly controlled by BSR1141. Of interest, it appears that BSR1141 positively regulates the expression of *virB2*, which encodes part of the critical VirB type IV secretion system (66), and this regulatory event may, in part, explain the attenuation of the *bsr1141* deletion strain. Indeed, mutation of *virB2* leads to attenuation of *Brucella* strains in cellular and animal models of infection, and thus, the decrease in *virB2* expression in the absence of BSR1141 may contribute to the decreased virulence of the *bsr1141* deletion strain (67). Nonetheless, more work is needed to fully define the regulatory activity of BSR1141.

Saadeh et. al took an alternative approach to find sRNAs in *B. suis* (51). This group utilized a co-immunoprecipitation approach with a chromosomally epitope-tagged (i.e., 3xFLAG tag) version of the RNA chaperone Hfq as bait to enrich for sRNAs, and following precipitation of the RNAs, strand-specific cDNA library generation and deep sequencing was performed. Instead of using prediction programs, this group mapped the transcriptomic data to the *B. suis* genome, concentrating on intergenic regions of the chromosomes. This technique led to the discovery of 33 candidate Hfq-associated sRNAs, of which 10 were confirmed using northern blot analysis. Target prediction analyses using TargetRNA determined putative regulatory associations for many of these sRNAs, but no validation was performed to authenticate the sRNA-target regulatory links (65). Overall, this work identified several novel Hfq-associated sRNAs, and additionally work is needed to define: 1 the regulatory activity of the sRNAs; and 2 the role of the sRNA in the physiology of *Brucella*.

Another strand-specific deep-sequencing approach focused on intergenic regions was used to identify 1321 sRNA candidates in *B. melitensis* (52). One candidate, BSR0441 was chosen for further characterization due to its predicted targets involvement in virulence, and the expression of BSR0441 was confirmed by RT-PCR. Deletion of *bsr0441* leads to decreased survival in murine macrophages at certain time points (i.e., 8-and 48-hours post-infection, but not 24-hours post-infection), indicating that BSR0441 may be important for the ability of *B. melitensis* to survive and replicate in macrophages. Regulatory targets of BSR0441 were predicted using TargetRNA2 (68); however, no direct assessment of BSR0441-target regulatory activity was performed. Rather, the expression of putative target mRNAs was analyzed during *B. melitensis* infection of macrophages and mice, and therefore, it is difficult to discern the impact of the sRNA on gene expression vs. the impact on the infection environment. In the end, further investigation is needed to define the regulatory mechanism of BSR0441.

In a study exploring RNaseIII, the investigators employed a high-throughput sequencing approach to identify 126 sRNAs in *B. melitensis* strain 027 (53). The goal of the study was to identify sRNAs that may be targeted by RNaseIII for regulatory events, and to this end, a representative sRNA, BM-sr00117, was confirmed via northern blot analysis. However, no further investigation involving this sRNA was performed. To further understand how BM-sr00117contributes to the biology of *Brucella*, more work is needed.

Xu et al. (54) predicted 14 sRNAs in *B. melitensis* M28 using two bioinformatic prediction programs, Softberry (http://www.softberry.com/) and Arnold (http://rssf.i2bc.paris-saclay.fr/toolbox/arnold/), that focused on conserved promoter and terminator elements within intergenic regions (54). Among these predicted sRNAs, seven were confirmed via RT-PCR and northern blot analyses, and these new sRNAs were named Bmsr for *Brucella melitensis* small RNA. One sRNA, Bmsr1 was studied further, and it was determined that deletion of *bmsr1* leads to reduced intracellular survival in RAW264.7 cells compared to the parental *B. melitensis* strain. Moreover, the *bmsr1* deletion strain was attenuated in a mouse model of infection, and taken together, these data indicate that Bmsr1 is important for the full virulence of *B. melitensis*. TargetRNA2 combined with iTRAQ proteomic analysis was used to develop of list of putative Bmsr1 regulatory targets, and interestingly, the *bmsr1* deletion strain showed lower levels of several VirB type IV secretion system proteins, as well as a reduction in levels of VjbR, a LuxR-type regulator. Both the VirB system and VjbR are well-document virulence factors in *Brucella* strains, and thus, dysregulation of these important virulence determinants likely plays a role in the attenuation of the *bmsr1* deletion strain (66, 69). While these findings are interesting and exciting, it is not clear how Bmsr1 is connected to the production of the VirB and VjbR proteins. Is this a direct regulatory event, or is this regulation indirect? Given the prominent roles played by the VirB system and VjbR, additional research is needed to characterize Bmsr1-mediated control of these systems.

Recent work investigating the endoribonuclease RNaseE uncovered determined that a sRNA named Bsr4, for *Brucella* small RNA, is dysregulated when RNaseE activity is compromised (55). Bsr4 had previously been identified in *B. melitensis* and in *B. suis* where it was called BSR1141 and BSnc118, respectively, and the authors designated the sRNA as Bsr4 as a means of clarifying the nomenclature of *Brucella* sRNAs (described in more detail in the following section) (49–51). Bsr4 was found to be highly abundant in a *rnaseE-trunc* mutant strain, indicating RNaseE aids in the degradation of Bsr4. Sheehan et al. (56) also confirmed that Bsr4 is dependent on the RNA chaperone Hfq for full stability, but *bsr4* is dispensable for *Brucella* infection, as the *bsr4* deletion strain exhibited no difference in the ability of the bacteria to survive and replicate in murine macrophages or to chronically infect mice. To date, the regulatory activity of Bsr4 remain uncharacterized.

### Contribution of sRNAs to *Brucella* biology and future directions

**Figure 2:**
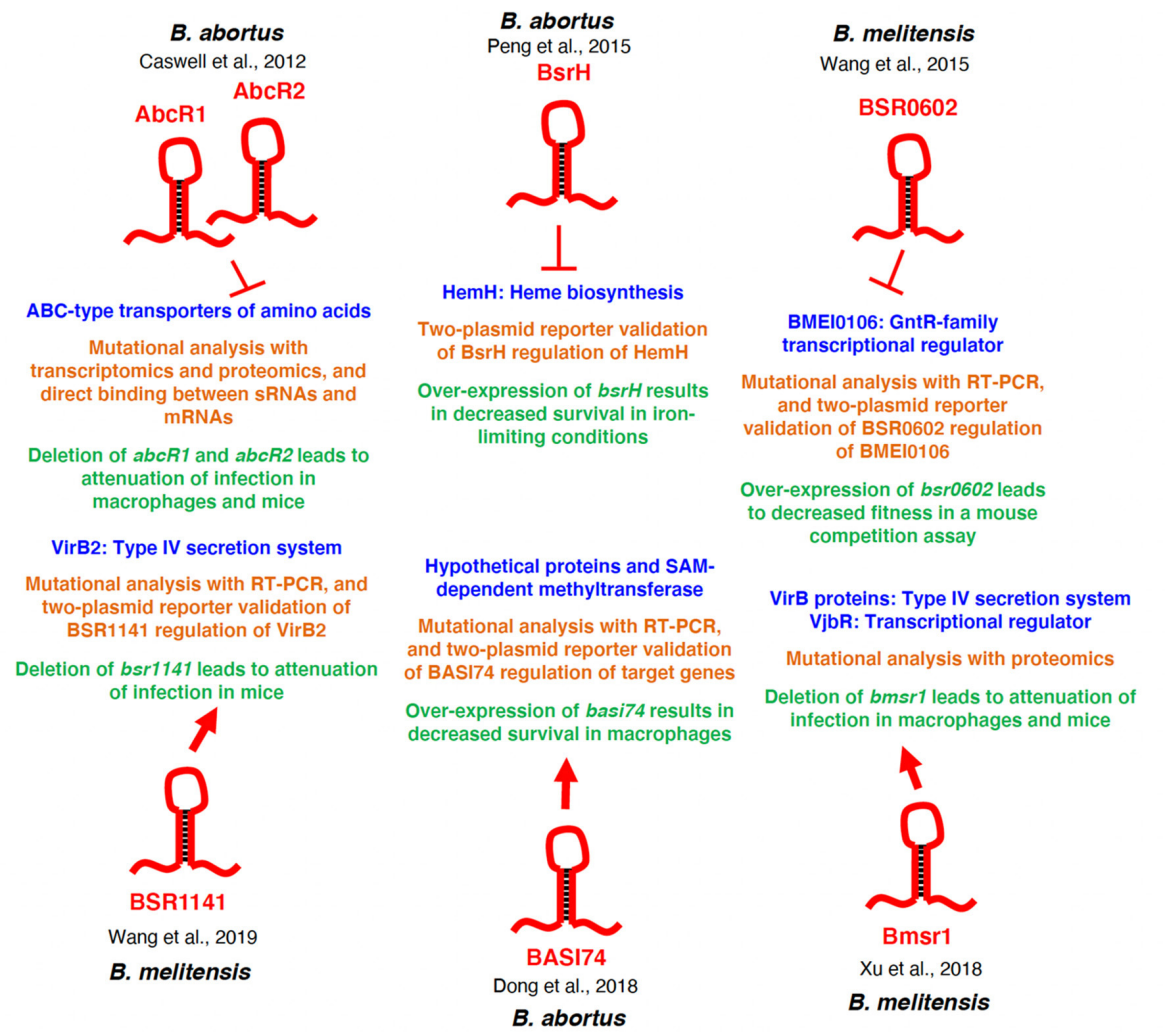
Regulatory overview of *Brucella* sRNAs. The specific sRNAs are depicted in red, and the regulatory targets/target systems are depicted in blue. The methods used to characterize the sRNA-target relationship are shown in orange, and the physiological consequences of deletion or over-expression of the sRNA is shown in green. Additionally, the *Brucella* species in which each sRNA was studied is indicated for each sRNA. Arrows indicate activation of expression, and perpendicular lines indicate repression of gene expression.

Even though many sRNAs that have been predicted in *Brucella* spp., only a few have been validated and further investigated. An overview of specific *Brucella* sRNAs and their regulatory targets is depicted in Figure 2. Many of these sRNAs are required for virulence and regulate transcripts that are key virulence factors of *Brucella*, and thus, sRNAs heavily contribute to the adaption process once inside the macrophage. However, many questions are still left unanswered regarding the authenticity of predicted regulatory targets of these sRNAs, and to answer this, experimental approaches need to be employed to validate *Brucella* sRNA target mRNA predictions.

In addition to the characterization of sRNA-mRNA regulatory activities, very little is known about how expression of *Brucella* sRNAs is controlled. What transcriptional regulators are orchestrating sRNAs levels in *Brucella*? What of these transcriptional regulators and/or the sRNAs themselves sensing and responding to in order to appropriately coordinate gene expression during infection? What are the mechanisms and processes of sRNA turnover in the brucellae? These are fundamental and extremely exciting questions that remain to be answered.

As highlighted in the previous paragraphs, there is a wide variation in nomenclature used in the *Brucella* sRNA field. This is completely understandable given that we all try our best to organize and collate data in a useful manner, but the lab-to-lab nomenclature of *Brucella* sRNAs is almost untranslatable. This can be extremely confusing for other researchers, even those very intimately connected to the field. As such, our group has developed a “Rosetta Stone of *Brucella* sRNAs” that we will continue to maintain as new sRNAs are identified and characterize, and the database is freely available on our laboratory website: http://caswelllab.com/. Our proposal is that designations be assigned as *Brucella* sRNA or “Bsr” regardless of the species in which the sRNA is identified. Given the high degree of similarity among *Brucella* species, it is highly likely that one sRNA in one species is also produced by other species (38–40). Admittedly, there may be sRNAs identified that are species-specific, and this should be noted accordingly and thoroughly investigated, as these findings could have implications in the speciation of *Brucella* and/or the natural host preference of *Brucella* strains. Nonetheless, the field will greatly benefit from an organized and systematic approach t o *Brucella* sRNA nomenclature, and we hope that others will join us in organizing a more clear and interpretable approach to designating sRNAs.

In the end, *Brucella* sRNAs represent an open frontier of research, full of exciting and presumably highly specialized regulatory mechanisms, and thus, much remains to be elucidated about sRNAs-mediated pathways in the brucellae.

The orientation of the sRNA gene is denoted with a red arrow.

Gene locus tags have been provided, and these locus tags are for three *Brucella* reference strains, even if a specific sRNA was identified in another *Brucella* strain. The reference strains and the web links to their genome information is as follows: *B. melitensis* 16M (https://www.ncbi.nlm.nih.gov/genome/943?genome_assembly_id=300462), *B. suis* 1330 (https://www.ncbi.nlm.nih.gov/genome/806?genome_assembly_id=300358), and *B. abortus* 2308 (https://www.ncbi.nlm.nih.gov/genome/520?genome_assembly_id=167411).

## Author contributions

KK and CC conceived the plan for the review. KK wrote the initial draft of the manuscript. MC and CC reviewed and edited the text. CC designed and constructed the figures. All authors contributed to the article and approved the submitted version.

## Funding

The National Institute of Allergy and Infectious Diseases has supported *Brucella* small RNA research in the Caswell laboratory (AI117648 and AI149124). MC is supported by the Animal Model Research for Veterinarians Program (T32OD028239) at Virginia-Maryland College of Veterinary Medicine.

## Conflict of interest

The authors declare that this work was carried out in the absence of commercial and financial relationships that could be seen as potential conflicts of interest.

## Publisher's note

All claims expressed in this article are solely those of the authors and do not necessarily represent those of their affiliated organizations, or those of the publisher, the editors and the reviewers. Any product that may be evaluated in this article, or claim that may be made by its manufacturer, is not guaranteed or endorsed by the publisher.
